# Microparticles controllable accumulation, arrangement, and spatial shaping performed by tapered-fiber-based laser-induced convection flow

**DOI:** 10.1038/s41598-017-14802-1

**Published:** 2017-10-30

**Authors:** Yu Zhang, Jiaojie Lei, Yaxun Zhang, Zhihai Liu, Jianzhong Zhang, Xinghua Yang, Jun Yang, Libo Yuan

**Affiliations:** 1 Key Lab of In-fiber Integrated Optics, Ministry Education of China, Harbin Engineering University, Harbin, China; 2 Guilin University of Electronic Technology, Guilin, China

## Abstract

The ability to arrange cells and/or microparticles into the desired pattern is critical in biological, chemical, and metamaterial studies and other applications. Researchers have developed a variety of patterning techniques, which either have a limited capacity to simultaneously trap massive particles or lack the spatial resolution necessary to manipulate individual particle. Several approaches have been proposed that combine both high spatial selectivity and high throughput simultaneously. However, those methods are complex and difficult to fabricate. In this article, we propose and demonstrate a simple method that combines the laser-induced convection flow and fiber-based optical trapping methods to perform both regular and special spatial shaping arrangement. Essentially, we combine a light field with a large optical intensity gradient distribution and a thermal field with a large temperature gradient distribution to perform the microparticles shaping arrangement. The tapered-fiber-based laser-induced convection flow provides not only the batch manipulation of massive particles, but also the finer manipulation of special one or several particles, which break out the limit of single-fiber-based massive/individual particles photothermal manipulation. The combination technique allows for microparticles quick accumulation, single-layer and multilayer arrangement; special spatial shaping arrangement/adjustment, and microparticles sorting.

## Introduction

The ability to arrange cells or microparticles into desired patterns is critical in numerous biological studies and applications such as microarrays^1,2^, tissue engineering^3,4^, and regenerative medicine^5,6^. Currently, several techniques and devices for micro manipulation have been implemented to supplement the conventional approaches: optical tweezers^7,8^ and optoelectronic tweezers^9,10^ offer single particle manipulation but have a limited capacity to trap plenty of particles simultaneously, owing to the strong focusing requirement, and thermo-/electro-/dielectrophoresis^11–17^ allows massive manipulation but lacks the spatial resolution necessary to manipulate individual particle. Several approaches have been proposed that combine both high spatial selectivity and high throughput simultaneously, including photothermal trapping^18^ and spatial patterning of plasmonic^19^, optofluidic^20,21^, and structured light landscapes^22,23^. However, those methods are complex, expensive, and difficult to fabricate. Therefore, we propose a method to combine the laser-induced convection flow method and fiber-based optical tweezers to manipulate and arrange microparticles. This method has the advantages of allowing massive particles manipulation and maintaining the necessary spatial resolution for individual particle manipulation. This approach is attractive because microparticles are attracted over long ranges by convection currents, thus allowing direct collection of particles. The laser-induced convection flow effects based on the optical fiber is more convenient for the manipulation along the vertical or depth direction, which has advantages over the micromanipulation based on the traditional optical lens^20–24^. The laser-induced convection flow method contain not only the batch manipulation of massive particles, but also the finer manipulation of special one or several particles, which breakouts the limit of single-fiber-based massive/individual particles photothermal manipulation. In addition, being different from the manipulation of thermos-optically^25,26^ and collective photothermal effect^27,28^, our method is much safer for most organisms. The thermos-optically and photothermal effect methods have to produce high temperature (>100 Celsius), which damage the living organisms in the solution. Moreover, compared with the light-induced hybridization of some special particles^29,30^, the laser-induced convection flow effects is more universal, working for most common particles. In other words, the samples are not necessary to have the characteristic of electronic, magnetic or plasmatic, our manipulation is applicable to most particles, from special to normal.

In this work, we combine the laser-induced convection flow method and the fiber-based optical trapping method to achieve massive/individual microparticles spatial shaping arrangement. Essentially, we combine a light field with a large optical intensity gradient distribution and a light-induced heated field with a large temperature gradient distribution to produce both the regular and special shape arrangements of microparticles. For the regular arrangement requirements, we may employ the thermal gradient distribution method, which is fast, simple and low-cost. For the special shape arrangements, we may switch to the fiber-based optical tweezers method.

## Results

### Principle of microparticles accumulation

**Figure 1:**
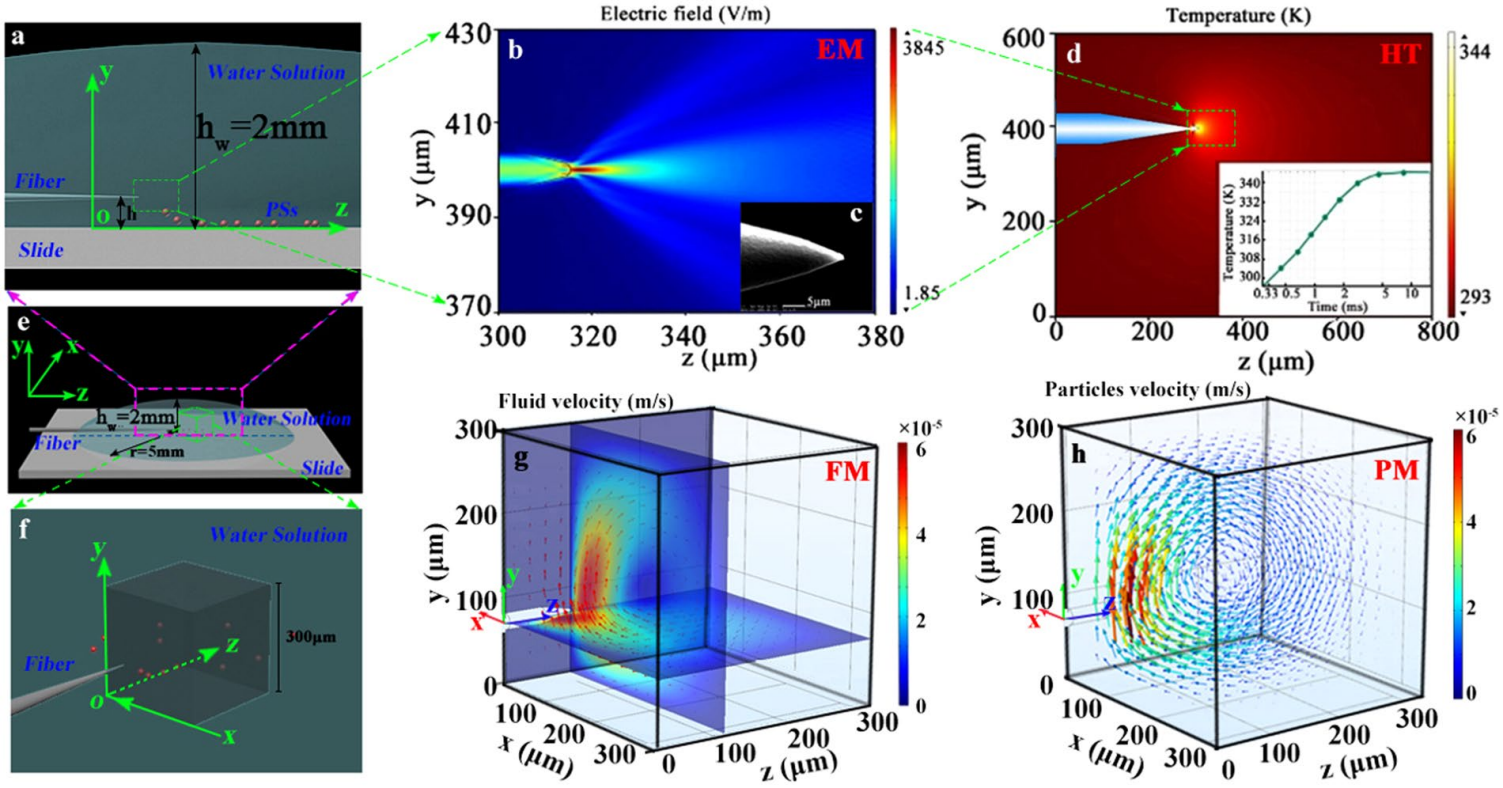
Simulated results of particles motions caused by the laser-induced convection. (**a**) 2-dimensional schematic diagram of the EM and HT simulated coordinate system; (**b**) Simulated results of the output light field; (**c**) SEM Image of the fiber tip; (**d**) Simulated results of the temperature field (the inset image shows the relaxation time); (**e**) 3-dimensional schematic diagram of the experimental configure; (**f**) Enlarged 3-dimensional schematic diagram to show the FM and PM simulated model; (**g**) Simulated results of the fluid velocity field (the arrows show the moving direction); (**h**) Simulated results of the particles velocity field (the arrows show the moving direction).

In this work, the laser-induced convection experiment is performed in a chamber with a drop of a water solution (~0.15 ml) on a slide, whose volume is about 2 mm in height and 10 mm in diameter (see Fig. 1e). Laser-induced convection flow arises because of the heat in the aqueous solution that results from the 1.48 μm laser power being absorbed strongly by water. This establishes a temperature gradient and produces the convection flow. The convection flow drives the microparticles towards the heat source and results in the accumulation and arrangement. As a key device, a fiber probe with a special shape tip (see Fig. 1c) is selected to integrate the light field and the thermal field. Thus, on the basis of the wavelength division multiplexing (WDM) technology, we achieve the independent-control of the light field and the thermal field.

The distributions of the light field, thermal field, fluid flow velocity field, and microparticles velocity field can be governed by a set of coupled partial differential equations describing the electromagnetic (EM), heat-transfer (HT), fluid mechanics (FM) and particles motion (PM) phenomena. (**1**) **EM**. We employ a normal commercial communication optical fiber (SMF-28, Corning©) with a special tapered tip (see Fig. 1c) in the experiment. The fabrication method of the fiber tip is described in the Method section. The fiber probe immerses in the aqueous solution and launches the laser power of 1.48/0.98 μm in wavelength. In order to describe the EM field, we employ the “Electromagnetic Wave, Frequency Domain” module in the Comsol software to simulate the output light field distribution (see Fig. 1b, the coordinate system is defined as shown in Fig. 1a and e). (**2**) **HT**. The heat transfer in the fluids solves for the equation as *ρC*_*p*_ **u**(**r**)·∇*T*(**r**) = ∇·(*k*∇*T*(**r**)) + *μ*_*α*_Φ(**r**), where *ρ* = 1 × 10^3^ kg/m^3^, is the fluid density, *C*_*p*_ = 4.2 × 10^3^J/(kg·K), is the specific heat capacity at constant pressure, *T*(**r**) is the temperature distribution, **u**(**r**) is the velocity vector of the heat transfer, *k* = 0.62 W/(m·K) is the thermal conductivity, *μ*_*α*_Φ(**r**) is the heat source caused by the laser power absorption, and *μ*_*α*_ = 26 cm^−1^, is the absorption coefficient^31^, Φ(**r**) (W/m^2^) is the total fluence rate, which can be obtained from the EM simulation results. We employ the “Heat Transfer in Fluid” module in the Comsol software to simulate the temperature field distribution in the solution (see Fig. 1d, the insect provides the thermal relaxation time, which is about 5 ms). (**3**) **FM**. We simulate the convection flow velocity field distribution by solving the equations of ∇·(*ρ* **u**_**f**_(**r**)) = 0 and *ρ*_0_(**u**_**f**_(**r**)·∇) **u**_**f**_(**r**) + ∇*p*(**r**) − η∇^2^ **u**_**f**_(**r**) = **g**(*ρ*(**r**) − *ρ*_0_), where **u**_**f**_(**r**) is the flowing velocity and *p*(**r**) is the pressure distribution, *η* = 1 × 10^−3^ Pa·s is the viscosity of the water, and **g** is the gravitational acceleration vector. The flow velocity pattern possesses a toroidal Rayleigh–Bénard flow (see Fig. 1g). Given that the buoyancy force is dependent on the spatial temperature distribution, the presence of the light source power strongly influences the peak convection velocity achieved in the system. In order to describe the toroidal flow, we build a 3-dimensional model by using the Comsol software (see Fig. 1f, which provides the position and volume of the model). We employ the “Laminar Flow” module in the Comsol software to simulate the fluid flowing tendency and velocity (see Fig. 1g, where the arrows provide the fluid flowing directions). (**4**) **PM**. We employ the “Particle Tracing for Fluid Flow” module in the Comsol software to simulate the moving velocity of the microparticles in the solution (see Fig. 1h). When the fluid flows, it will drive the immersed microparticles moving. The microparticles motion can be described as, **F** = *d*(*m*_*p*_ **v**)/*dt* = *m*_*p*_(**u**_**f**_ − **v**)/(*τ*_*p*_ *S*), where *τ*_*p*_ = *ρd*_*p*_^2^/(18*η*), and *S* is a constant, *m*_*p*_ is the particle mass, *d*_*p*_ is the particle diameter, **v** is the particle moving velocity.

### Microparticles accumulation and regular arrangement

**Figure 2:**
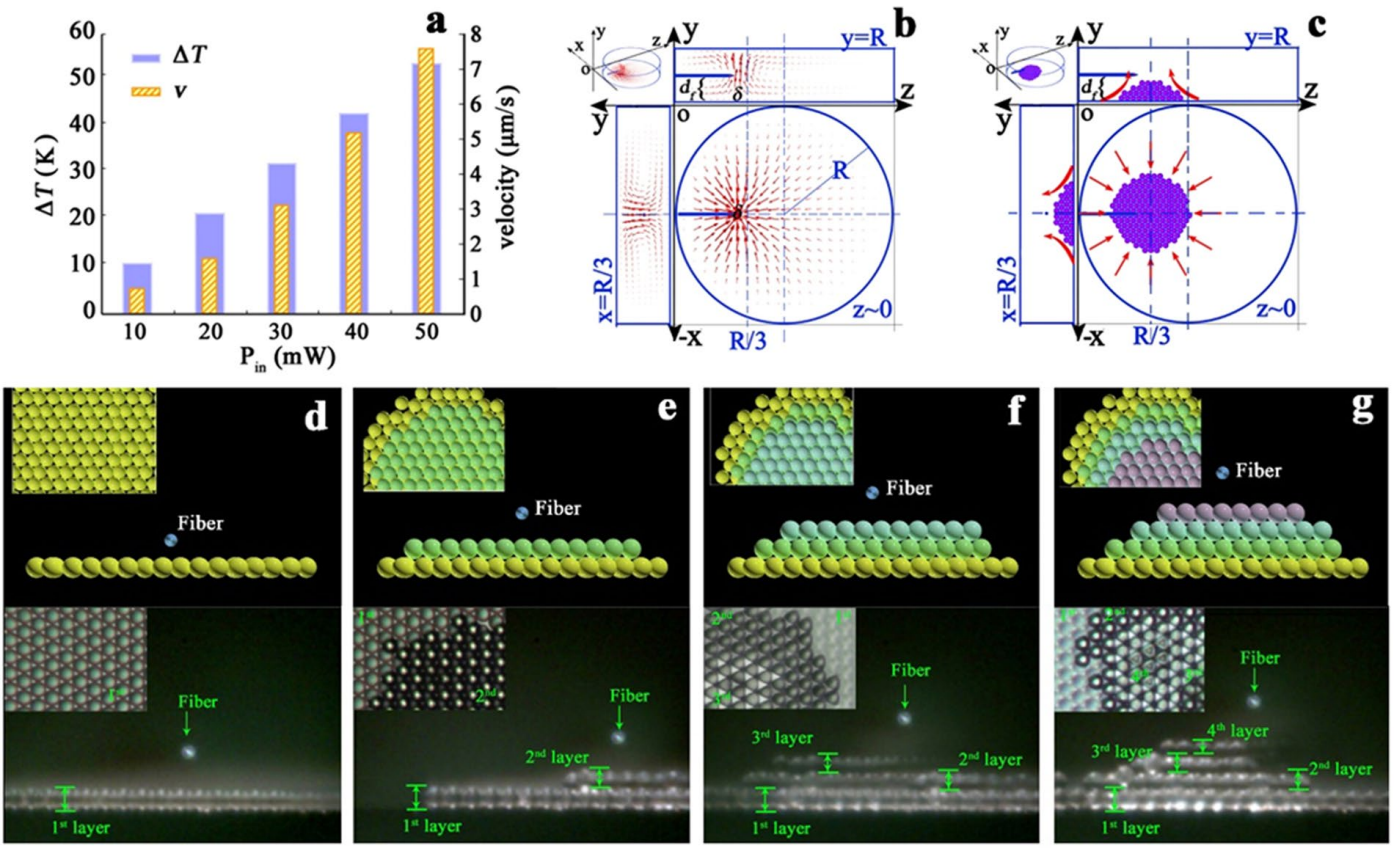
Experimental results. (**a**) Maximum fluid velocity and temperature increase for various light source power; (**b**) The simulated results of the PSs moving speed distribution, the red arrows represent the PSs moving speeds; (**c**) The sketch diagram of the laser-induced convection flow forces keeping the PSs arranging compactly and stably; (**d,e**) Schematics and images of the microparticles multilayer arrangement from the side view (the small images in the upper-left corner of each image are seen from the top view.

When the laser source (1.48 μm in wavelength) power is smaller than a critical value (here is 53 mW, the explanation of the critical laser power is provided in the Method section), the microparticles (polystyrene spheres, PSs. The diameter of the PS is 10 μm) in the aqueous solution achieve regular accumulation and arrangement in a single layer on the bottom of the solution. The trapped PSs organize themselves into a tightly packed hexagonal assembly. We measure the accumulating velocity **v** of PSs with different input laser power, *P*_in_ = 10, 20, 30, 40, 50 mW. The results are summarized in Fig. 2a. Given that **v** and Δ*T* scale linearly with the input power over the entire range of Δ*T* = 0 to Δ*T* = 51 K, the larger the laser power *P*_in_, the larger the accumulating velocity **v** (see Supplementary Movie 1). When the laser power is 50 mW, more than 600 PSs accumulate within 180 s. Thus, the average accumulating speed is about 3.33 ± 0.03/s.

### Microparticles multilayer arrangement

When the PSs fall in the effective convection area, they begin to fill up the potential well from the bottom until the bottom of the potential well is fully occupied. An increase in the potential well depth results in an increase in the number of PSs trapped in the area. When we move the fiber probe upwards slightly, which corresponds to adding the effective trapping volume, the PSs stack layer by layer vertically into a three-dimensional tower cluster [see Fig. 2e–h and Supplementary Movie 2]. The stack has 4 layers. From the top view, we may distinguish the PSs multilayer arrangement due to the refractive index difference performed by the multilayer PSs superposition. From the side view, the multilayer arrangement is visible clearly. According to the Fig. 2b and c, which provides the convection flow currents distribution in the three-dimensional coordinate system. In the -xoz plane, the flow currents are towards the position of δ (labeled in Fig. 2b), where the flow currents turn upwards from the bottom of the substrate, being along the y-axis direction. On the bottom of the substrate, the pushing forces from all direction towards the position of δ will take along the PSs on the bottom to accumulate around δ. Due to the spherical shape of the PSs, they are packed compactly in the hexagonal shape. The pushing forces are regarded as the fence around the accumulated PSs, which keeps the PSs forming compact and stable arrangement. When the PSs finish the filling and arrangement of the 1^st^ layer, there are still plenty of PSs in the solution, the pushing forces still exist, which causes the second layer arrangement of PSs. Similarly, the pushing forces can still be regarded as the fence around the two-layer PSs, which keep the PSs forming the stable arrangement. Similarly available, the laser-induced convection performs the arrangement of PSs in the third and fourth layers and more. In addition, the number of the layers is also determined by the depth of the fiber probe *d*_*f*_ (labeled in the figure below).

### Microparticles special-spatial-shape arrangement

**Figure 3:**
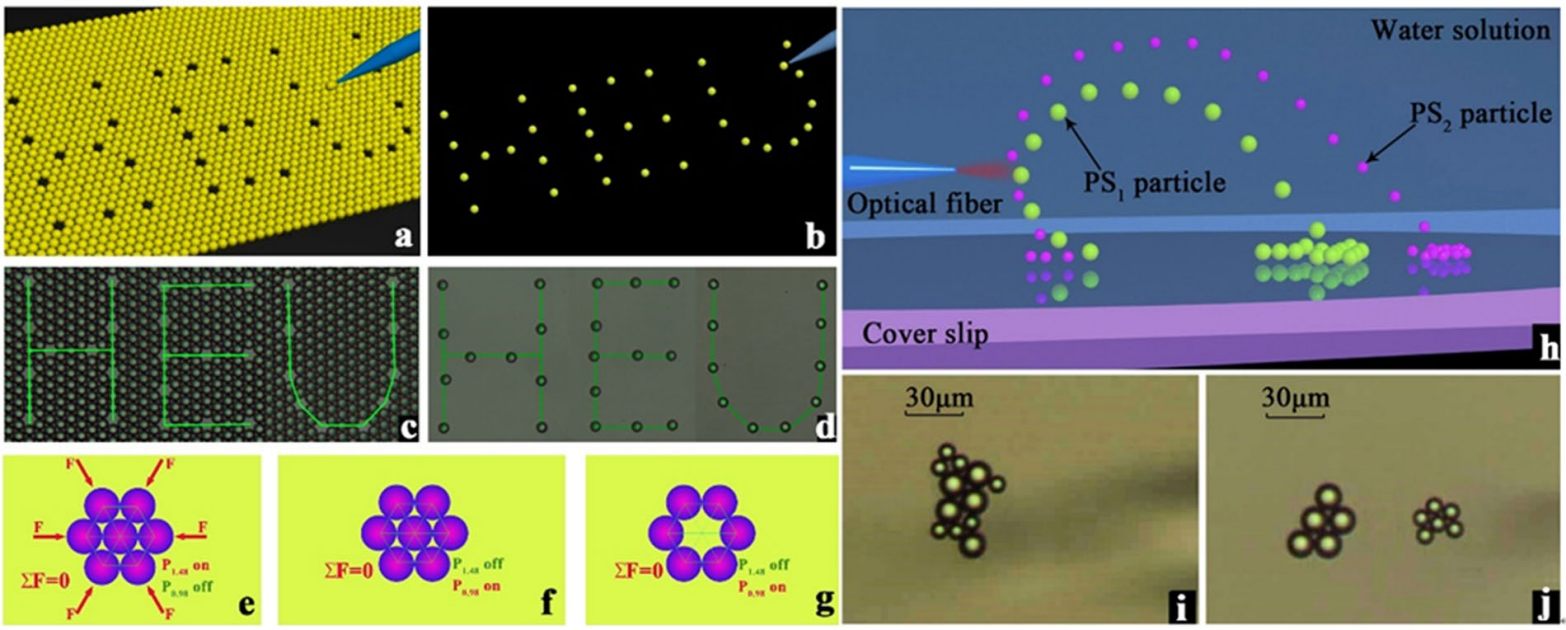
Experimental results. (**a**), (**b**) Schematics and (**c**), (**d**) Images of the especially spatial shaped arrangement of the microparticles combined with the optical tweezers technology; (**e**–**g**) Schematics of the net forces exerting on an arranged unit (**j**) Schematics of microparticles sorting principle and (**k**), (**l**) images of the microparticles sorting.

We may arrange the PSs in a special shape by the switch to the fiber-based optical tweezers working mode. After the PSs finishing the regular arrangement in the single layer, we employ the same fiber probe, switching the laser source wavelength to the 0.98 μm, to trap individual PS at certain locations. Thus, we may achieve the vacancy arrangement of 31 positions in the shape of letters “H”, “E” and “U” (see Fig. 3a and c). Additionally, we may arrange the picked-out PSs in the special shape of letters “H”, “E” and “U” (see Fig. 3b and d) in other positions. In the practical experiment, we employ a glycerin-water solution (volume ratio 1:4) to reduce the perturbation of PSs in the chamber. After the accumulation and arrangement of the PSs, which are trapped into a tightly packed hexagonal shape. The Fig. 3e provides the schematics of the arrangement unit, the net force exerting on the unit is zero. When we turn off the power of 1.48 μm laser source and turn on the power of 0.98 μm laser source, due to the absence of the heat absorption, the temperature of the solution drops back to the room temperature, the temperature gradient in the solution is very small. The interaction forces of the PSs in an arrangement unit is zero (see Fig. 3f). Therefore, the PSs will keep the hexagonal shape arrangement. In other words, the particle will keep the position unchanged. When we pick up the particle in the center of the unit, the interaction forces of the leaving PSs are still zero, therefore, the pickup manipulation will not affect the arrangement of the PSs (see Fig. 3g). The neighbor particles are not affected by the removed particle.

### Microparticles sorting

When the PSs of different sizes are in the same convection flow field, the accumulating velocity is determined by the sizes of the PSs. The larger the PS diameter, the smaller the vertical component of the accumulating velocity. When the laser source power is 60 mW, the PSs may exhibit the motion along the y-axis direction, achieving the toroid flow (see Fig. 1h). If we move the fiber probe downwards, the toroid flow shape will not be a closed loop (see Fig. 3h). When the PS_1_ (with a diameter of 10 μm) and PS_2_ (with a diameter of 15 μm) are in the solution, the accumulating velocities of the PS_1_ and PS_2_ are different, achieving the sorting of PSs (see Fig. 3i and j). According to the schematic diagram, the particles of different sizes flow in different trajectories, if we move the fiber probe downwards, we then achieved sorting of the microparticles with different *r*_*p*_ values.

## Discussions

**Figure 4:**
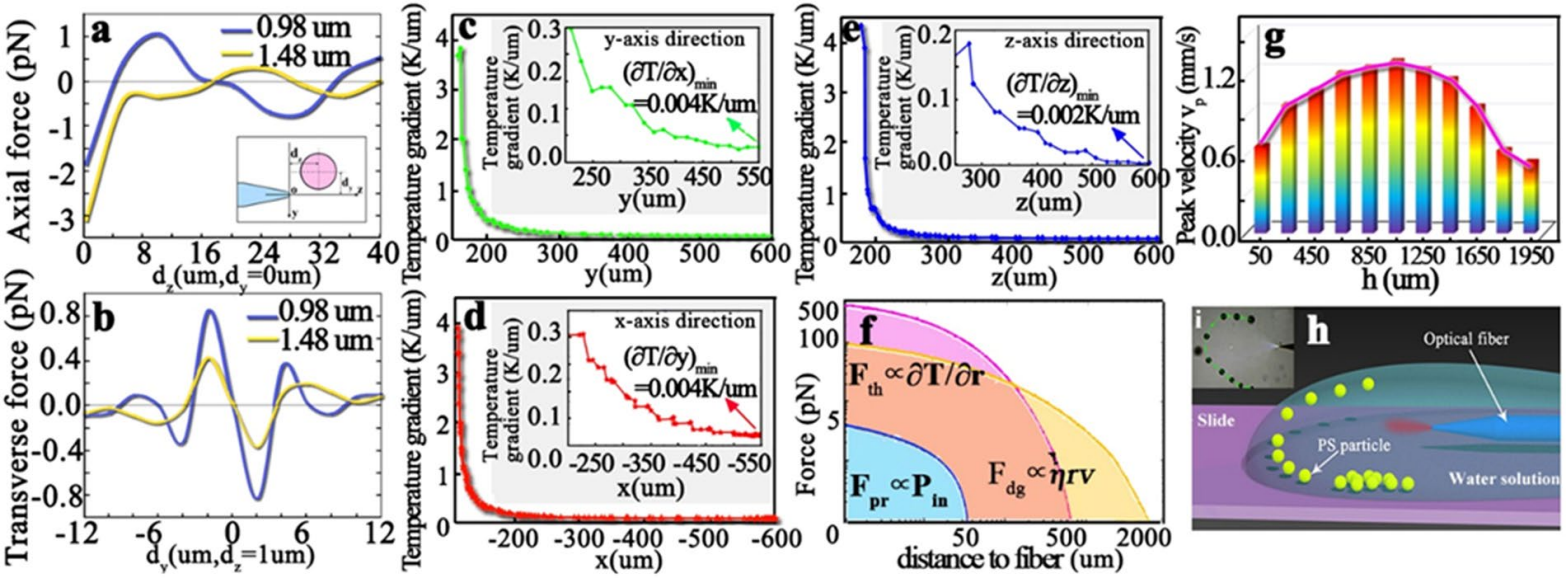
Forces and velocities. Simulated results of 1.48 μm and 0.98 μm laser axial trapping force (**a**) and transverse trapping force (**b**), where d_z_ and d_y_ are the axial and radial distances between the fiber tip and the PS center, which are shown in the inset in (**a**), the refractive index of PS is 1.59 and the power of the incident laser is 1 mW; Simulated results of the temperature gradient distribution produced by the laser-induced heated field along the x-axis direction (**d**), y-axis direction (**c**), and z-axis direction (**e**); the insects in them provide the enlarged information of the temperature gradient distribution in different ranges; Here the spatial coordinate (see Fig. 1g) of the fiber tip is (0,150,180), and unit is μm; (**f**) Comparison among the optical radiation pressure force F_pr_, the thermophoretic force F_th_, and the dragging force F_dg_ produced by the solution convection flow. The coordinate in the (**f**) is logarithmic; Schematic (**h**) and Image (**i**) of the semicircular shape arrangement of the microparticles.

In addition to the thermal effect, the 1.48 μm laser source produces the optical radiation pressure forces. Figure 4a and b provides the simulated results of the axial (*z-*axis direction) trapping forces and the transverse (*y*/*x-*axis direction) trapping forces produced by the 1.48 μm laser source. (The optical force calculated method is described in the Methods section). As shown in Fig. 4a and b, the axial force is in the magnitude order of ~pNs/mW, which means when the incident laser power is 1 mW, the optical trapping forces are in the magnitude order of 1pN. However, in the practical experiment, the strong absorption of the 1.48 μm laser by the water causes the violent vibration of the water near the fiber tip, which prevents the stable trap. In addition, according to the simulated results, the effective trapping range of the radiation pressure forces is <20 μm, which is too short to achieve the aggregation of thousands of PSs. Therefore, the radiation pressure trapping forces produced by the 1.48 μm laser source don’t contribute the aggregation manipulation of microparticles.

When the 1.48 μm laser source is off and 0.98 μm laser source is on, the effect of laser-induced convection flow disappears, there only exists the radiation pressure force produced by the 0.98 μm laser source. Figures 4a and b also provide the simulated results of the axial and transverse trapping forces produced by the 0.98 μm laser. Compared with the forces produced by the 1.48 μm laser, the optical trapping forces produced by the 0.98 μm laser are larger. The 0.98 μm laser source is suitable to trap and manipulate the PSs. We may employ the fiber probe to pick up the PSs on some special positions after they aggregate and arrange, or place the picked-up PSs on the special positions to achieve the special spatial shaping as seen in Fig. 3a–d.

Microparticles thermophoresis is a non-equilibrium cross-flow effect between mass and heat transport. When a colloidal suspension is placed in a temperature gradient field, the microparticles will be arrived by a thermophoretic force and perform a steady thermophoretic drift velocity^32,33^. The thermophoretic force is proportional to the temperature gradient (see Method section). Figures 4c–e provide the temperature gradient distributions of the fluid along the x-, y-, and z-axis directions respectively. The temperature gradient is several K/μm near the fiber tip, therefore, the thermophoretic force is in the magnitude order of ~100pN, and the thermophoretic velocity is in the magnitude order of ~1 mm/s. However, this area is small (the distance *L* < 50 μm), the temperature gradient decline strongly (being about 1/10) when the distance between the fiber tip is >50 μm. When the PSs is far away from the fiber tip (*L* > 500 μm), the temperature gradient reduces to be several K/mm, therefore, the thermophoretic force is in the magnitude order of ~0.1pN, and the thermophoretic velocity is in the magnitude order of ~1 μm/s. Compared with the experimental results, the PSs accumulating velocity is in the magnitude order of ~mm/s within the range of ~1 mm. Therefore, when the PSs are far away from the fiber probe (>500 μm), the laser-induced convection flow makes a contribution to microparticles accumulation, when the PSs are near the fiber probe (<50 μm), the thermophoretic plays a leading role. In the middle area (50 μm < L < 500 μm), both two forces exert on the PSs.

Figure 4f provides the comparison of the optical radiation pressure force **F**_pr_, the thermophoretic force **F**_th_, and the dragging force **F**_dg_, produced by the solution convection current flow. **F**_pr_, is determined by the incident 1.48 μm laser source power P_in_. If the heating absorption of the solution does not disturb the optical radiation pressure, the 1 mW laser can produce about 1pN optical radiation pressure force. The effective range of **F**_pr_ is short, within 50 μm (see Fig. 4a and b). **F**_th_ is determined by the gradient distribution of the temperature field. As seen in Fig. 4c–e, the temperature gradient declines strongly, which is scaled in the form of exponential function. According to the temperature gradient experimental results, we see that the **F**_th_ declines from 500pN to 0 when the distance to the fiber tip increases from 0 to 500 μm. **F**_dg_ is determined by the relative speed **v**_p_, which is the difference between the PSs moving speeds and the solution current flow speeds. In the range of 0–2000 μm, the **F**_dg_ declines from the 100pN to 0. Compared with **F**_th_, the declining trend of the **F**_dg_ is slower. In summary, when the PSs are far away from the fiber probe (500 μm < L < 2000 μm), the laser-induced convection flow makes a contribution to microparticles accumulation, ensuring the PSs transported towards the fiber probe; when the PSs move near to the fiber (0 < L < 50 μm), the thermophoretic plays a leading role. In the middle area (50 μm < L < 500 μm), both forces, **F**_th_ and **F**_dg_ exert on the PSs.

The thickness of the water layer is about 2 mm, and we investigate the relationship between the fiber probe depth and the PSs peak velocity. According to Fig. 4g, the PSs peak velocity reaches the maximum, of 1.24 mm/s when the depth *h* (labeled in Fig. 1a) of the fiber probe is near the half of the water layer thickness (~1 mm). The smaller the distance between the fiber probe and the water top (bottom) interface, the smaller the PSs peak velocity.

When an interface exists between a liquid (water droplet) and a solid (glass slide), there exists a contact angle due to the wettability and the surface tension. Therefore, the edge of the water droplet is arc-shaped (see Fig. 4h and i). When the fiber tip is near the interface of the water and air, the scatting force and the convection force pull the particles from the substrate, and the microparticles move along the inner surface of the interface baseline, realizing the semicircular shape arrangement, as seen in Fig. 4h and i and Supplementary Movie 3.

## Methods

### Two-step method to fabricate the fiber tip

We develop a two-step method to fabricate the fiber tip. The two steps include chemical etching and discharged current fusion molding. Step 1: a selective chemical etching procedure is used. The chemical etching solution is composed of 48% hydrofluoric acid (HF), 40% ammonium fluoride (NH_4_F), and deionized distilled water. The volume ratio of the three solutions is 1.5:1:1^34^. Here, we chose 1-bromodecane as the protective liquid layer^35^, which is very important for the final profile of the fiber tip. After etching in solution for 60 min at room temperature, the cone angle of the tapered fiber tip is 30°. Step 2: a special discharge current fusion molding procedure is used. The single arc discharge, with a weak fusion current and a short fusion time, is generated from a commercial fusion splicer (Fujikura FSM-60s) and used to fuse and mold the fiber tip (prepared in step 1) into a special tapered-tip profile.

### Temperature measurement

The temperature measurement is based on measuring the fluorescence efficiency of rhodamine B (0.1 mM solution), whose temperature dependence has been well documented^36^. In the measurements, a 532 nm green laser is used to excite the fluorescence. A spectrometer (Ocean Optics 2000) is used to collect the fluorescence spectrum. This calibration is repeated three times to determine the temperatures when illuminated by 1.48 μm laser with the power of 50 mW.

### Calculation of optical trapping force

We employ the COMSOL software to simulate output light field intensity distribution and calculate the optical radiation pressure force. We use the Maxwell stress tensor^37^, which is provided by the electromagnetic wave, frequency domain interface in the COMSOL software, to derive the optical forces exerting on the PSs^38^. The stress tensor approach is exact and does not rely on any assumption regarding the nature of the field (whether evanescent or propagating) or of the objects. Assuming a steady-state condition, the net radiation force **F** on the particle can be determined by integrating the dot product of the outwardly directed normal unit vector $\hat{{\bf{n}}}$ and Maxwell’s stress tensor $\overleftrightarrow{{\bf{T}}}$ over a surface enclosing the particle, as ${\bf{F}}=\mathop{\oint }\limits_{s}\hat{{\bf{n}}}\cdot \overleftrightarrow{{\bf{T}}}dS$, where 〈 〉 represents a time average. For steady-state optical conditions, the Maxwell’s stress tensor can be expressed as, ${T}_{ij}=\frac{1}{2}{\rm{Re}}[\varepsilon {{\bf{E}}}_{{\boldsymbol{i}}}{{\bf{E}}}_{{\boldsymbol{j}}}+\frac{1}{\mu }{{\bf{B}}}_{{\boldsymbol{i}}}{{\bf{B}}}_{{\boldsymbol{j}}}-\frac{1}{2}(\varepsilon {|{\bf{E}}|}^{2}+\frac{{|{\bf{B}}|}^{2}}{\mu })\overleftrightarrow{{\bf{I}}}]$. Therefore, we may calculate the optical radiation pressure exerting on the PSs. Here, we employ the normal calculated method to estimate the magnitude of order of the optical radiation pressure. In the COMSOL software, the light radiation pressure exerting on the particles can be calculated by:${F}_{{\rm{x}}}=\mathop{\oint }\limits_{{\rm{s}}}({\rm{ewfd}}.{\rm{dnTx}}){\rm{ds}}$, ${{\rm{F}}}_{{\rm{y}}}=\mathop{\oint }\limits_{{\rm{s}}}({\rm{ewfd}}.{\rm{dnTy}}){\rm{ds}}$, and ${{\rm{F}}}_{{\rm{z}}}=\mathop{\oint }\limits_{{\rm{s}}}({\rm{ewfd}}.{\rm{dnTz}}){\rm{ds}}$, where ${\rm{ewfd}}\mathrm{.dnTx},\,{\rm{ewfd}}\mathrm{.dnTy},\,\mathrm{and}\,\mathrm{ewfd}{\rm{.dnTz}}$ are the downward x, y, z component of Maxwell’s stress tensor. We have verified that the calculated result is consistent with the analytical solution of MIE.

### Calculation of thermophoretic force

For a large particle (*r*_*p*_ ≫ *λ*_0_, *r*_*p*_ is PS radius, and *λ*_0_ is the mean free path of the fluid), the thermophoretic force can be expressed as **F**_th_ = −9*πr*_*p*_ *η* ^2^ *H*∇*T*(**r**)/(*ρT*), where *H* = (*k/k*_*p*_ + 2.2*λ*_0_ */r*_*p*_)/[(1 + 3*λ*_0_ */r*_*p*_)(1 + 2*k/k*_*p*_ + 4.4*λ*_0_ */r*_*p*_)], is a coefficient that accounts for the differences in thermal conductivity and size effect, which can be further reduced down to the ratio of the conductivities. Therefore, the thermophoretic force can be described as **F**_th_ = −9*πr*_*p*_ *η* ^2^ *k*∇*T*(**r**)/[*ρT(k*_*p*_ + *2k)*].

### Critical power

The elevated temperature near the fiber probe reduces the density of the water, resulting in buoyancy–driven flow as the fluid in this region rose. As expected with convection currents, flow velocities are determined primarily by the fluid temperature, which in turn is controlled by the light source power. When we add the light source power, the currents form a circular vortex in which microparticles are trapped. The convection solution causes the microparticles to accumulate. Within this volume, the microparticles flow vertically upwards, resulting in outward flow at the top view of the fluid. As observed in three–dimensions, the flow pattern is toroid centered. The flow pattern is similar to that in a single Rayleigh–Bénard cell in which the convection rolls are radially oriented. Under the action of gravity and viscosity alone, the flow of the microparticles problem in a flow is given by the equation of **v**_*p*_ = **v**_**r**_ + 2(*ρ* − *ρ*_*p*_)*r*_*p*_^2^ **g**/9, where **v**_**r**_ = **u**_f_ − **v**, is the relative velocity of the particle compared to the fluid flow, *ρ*_*p*_ is the mass density of the particle. For an approximately spherical particle with radius *r*_*p*_. The third term on the right-hand side is the gravitational term, proportional to (*ρ* − *ρ*_*p*_) and *r*_*p*_^2^. We decompose the **v**_*p*_ in the *y*-, *x*-and *z*- axis directions: v_*py*_ = v_*ry*_ − 2(*ρ* − *ρ*_*p*_)*r*_*p*_^2^g/9, v_*px*_ = v_*rx*_ and v_*pz*_ = v_*rz*_. When v_*py*_ < 0, the PSs will move in a 2D space, thus resulting in microparticles accumulation and regular arrangement. When v_*py*_ > 0, the PSs will move in a 3D space, resulting in microparticles toroid flow. The v_*py*_ is determined by the incident laser power, therefore, there exists a critical laser power P_c_, when incident laser power P_0_ > P_c_, the PSs may move in the 3D space; when incident laser power P_0_ < P_c_, the PSs have to move in the 2D space.

### Effects of evaporation

We employ a coverslip as the substrate, whose size is 1 cm×1 cm × 50 μm (length × width × height). The volume of the solution is ${\rm{\pi }}\times {(5{\rm{mm}})}^{2}\times 2\,{\rm{mm}}=0.157\,{\rm{ml}}$. The diameter of the fiber is 125 μm. The effective heating volume of the fiber probe is $\,{\rm{\pi }}\times {(62.5{\rm{\mu }}{\rm{m}})}^{2}\times 200\,{\rm{\mu }}{\rm{m}}/3=8.2\times {10}^{-7}\,{\rm{ml}}$, which is too small than the solution volume. The thermal diffusivity of water is $k/({\rm{\rho }}{c}_{p})=0.143\,\,{{\rm{mm}}}^{2}/s.{\rm{T}}$he results indicate that when the distance away to the heating source increases from 0 to 500 μm, the temperature gradient reduces to 1% of the initial value. Therefore, compared with the fiber probe, the solution is adequate for the laser-induced convection flow manipulation. In the practical experiment, we adjust the power of the 1.48 μm laser source from 30 mW to 50 mW, the duration time is 0.5 h to 1 h, which is long enough for most manipulations. The experimental results show that the average evaporated volume is 5 ± 0.1%, the left solution volume is still adequate. Therefore, we neglect the evaporation effect.

## Electronic supplementary material

Supplementary Information

Supplementary movie 1

Supplementary movie 2

Supplementary movie 3
