# Supplementary material for: Microparticles controllable accumulation, arrangement, and spatial shaping performed by tapered-fiber-based laser-induced convection flow
Source: Sci Rep. 2017 Oct 30;7:14378. doi: 10.1038/s41598-017-14802-1 (PMC5662569; doi:10.1038/s41598-017-14802-1)
Supplement: Supplementary file 1 — Supplementary Information [file 41598_2017_14802_MOESM1_ESM.doc]

**Supplementary Information**

**Microparticles controllable accumulation, arrangement, and spatial shaping performed by tapered-fiber-based laser-induced convection flow**

Yu Zhang, Jiaojie Lei, Yaxun Zhang, Zhihai Liu, Jianzhong Zhang, Xinghua Yang, Jun Yang, Libo Yuan

Key Lab of In-fiber Integrated Optics, Ministry Education of China,

Harbin Engineering University;

Correspondence and requests for materials should be addressed to Zhihai Liu

(e-mail:zhihai@vip.sina.com).

Content

**Supplementary Movie 1:  The micro particles accumulate.**


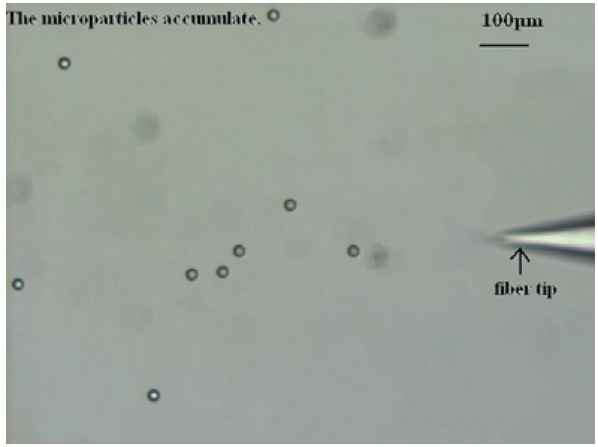


Supplementary Figure S1:  Video screenshot of movie 1.

**Supplementary Movie 2:  The micro particles arrange in multilayer.**


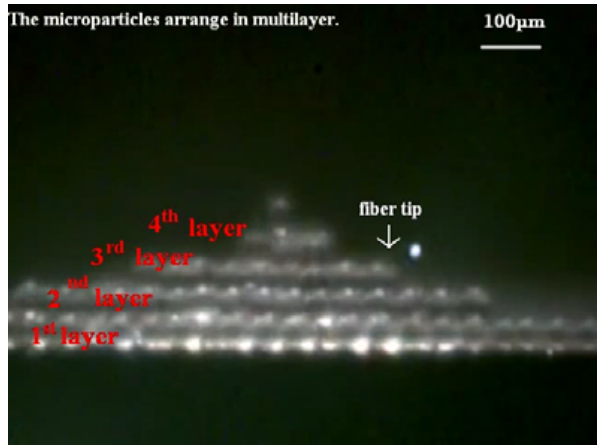


Supplementary Figure S2:  Video screenshot of movie 2.

**Supplementary Movie 3:  The micro particles arrange in semicircular shape.**


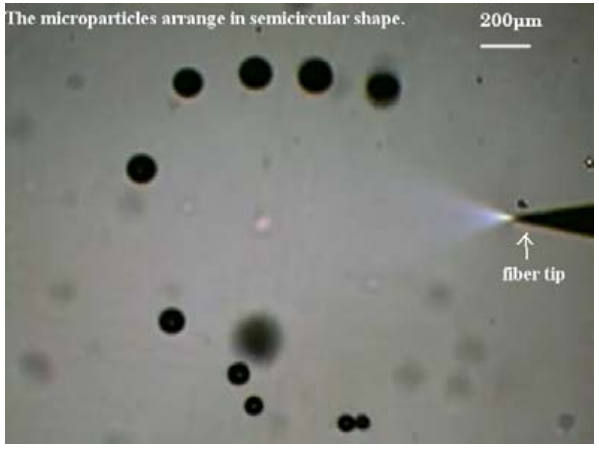


Supplementary Figure S3:  Video screenshot of movie 3.
